# The complete chloroplast genome of *Diplodiscus trichospermus* and phylogenetic position of Brownlowioideae within Malvaceae

**DOI:** 10.1186/s12864-023-09680-z

**Published:** 2023-09-26

**Authors:** Mingsong Wu, Liu He, Guangyao Ma, Kai Zhang, Haijian Yang, Xinquan Yang

**Affiliations:** 1 Hainan Provincial Key Laboratory of Resources Conservation and Development of Southern Medicine, Hainan Branch of the Institute of Medicinal Plant Development, Chinese Academy of Medical Sciences & Peking Union Medical College, Haikou, 570311 China; 2 Ministry of Education Key Laboratory for Ecology of Tropical Islands, College of Life Sciences, Hainan Normal University, Haikou, 571158 China

**Keywords:** Plastome, Phylogeny, Malvales, RSCU, SSRs, Palindromic sequence

## Abstract

**Background:**

Malvaceae is an economically important plant family of 4,225 species in nine subfamilies. Phylogenetic relationships among the nine subfamilies have always been controversial, especially for Brownlowioideae, whose phylogenetic position remains largely unknown due to the lack of samples in previous analysis datasets. To greatly clarify the phylogenetic relationship of Malvaceae, we newly sequenced and assembled the plastome of *Diplodiscus trichospermus* taxonomically located in Brownlowioideae, and downloaded the allied genomes from public database to build a dataset covering all subfamily members of Malvaceae.

**Results:**

The annotation results showed that the plastome of *Diplodiscus trichospermus* has a typical quadripartite structure, comprising 112 unique genes, namely 78 protein-coding genes, 30 tRNA genes and 4 rRNA genes. The total length was 158,570 bp with 37.2% GC content. Based on the maximum likelihood method and Bayesian inference, a robust phylogenetic backbone of Malvaceae was reconstructed. The topology showed that Malvaceae was divided distinctly into two major branches which were previously recognized as Byttneriina and Malvadendrina. In the Malvadendrina clade, Malvoideae and Bombacoideae formed, as always, a close sister clade named as Malvatheca. Subfamily Helicteroideae occupied the most basal position and was followed by Sterculioideae which was sister to the alliance of Malvatheca, Brownlowioideae, Dombeyoideae, and Tilioideae. Brownlowioideae together with the clade comprising Dombeyoideae and Tilioideae formed a sister clade to Malvatheca. In addition, one specific conservation SSR and three specific palindrome sequences were observed in Brownlowioideae.

**Conclusions:**

In this study, the phylogenetic framework of subfamilies in Malvaceae has been resolved clearly based on plastomes, which may contribute to a better understanding of the classification and plastome evolution for Malvaceae.

**Supplementary Information:**

The online version contains supplementary material available at 10.1186/s12864-023-09680-z.

## Background

Comprehensive and robust phylogenetic trees can advance our understanding of life origin, species differentiation and evolutionary process [1]. Complete chloroplast (cp) genomes usually inherited maternally have a moderate nucleotide substitution rate [2], and provide variation-rich nucleotide sequences compared with a few plastid or nuclear DNA markers, which have been widely used for plant phylogeny reconstruction, estimating divergence and generating genetic markers in recent years [1, 3–7]. Predominantly, the whole cp genomes contain 5–130 genes and its size ranges from 11 kb [8] to 240 kb (Accession: NC_031206 unpublished) in land plants, and generally exhibits a typical quadripartite structure consisting of two inverted repeats (IRs), one small single copy (SSC) and one large single copy (LSC) [9].

Malvaceae, the largest family in Malvales, incorporates the former separate families Malvaceae s.s., Bombacaceae, Sterculiaceae, and Tiliaceae [10]. It comprises 4,225 species in 244 genera in nine subfamilies [10, 11]. They are distributed more abundantly in tropical and subtropical regions, and were found all over the world except from the Arctic, the Antarctic, and the Gobi Desert [11]. Malvaceae, several members of which are widely used in agriculture, forestry, and horticulture, is an economically important plant family within the order Malvales in rosids. The economic importance includes herbal medicines [12, 13], fibers [14], gums [15, 16], fruits [17], vegetables [18–22], oils [23], beverages [14], timbers [24, 25], and numerous ornamental cultivars [26].

In traditional circumscription, Tiliaceae, Sterculiaceae, Bombacaceae, and Malvaceae s.s. were recognized as the "core Malvales", and the close relationship among these families was generally recognized [27, 28]. The first phylogenetic study focused on this group based on morphological features showed that only Malvaceae s.s. is likely monophyletic, and the other three families are paraphyletic or polyphyletic. Therefore, the "core Malvales" were proposed to be recognized at the familial level, i.e., Malvaceae s.l. [29]. This taxonomic treatment was supported by subsequent both morphological and molecular studies [10, 30–34]. Based on molecular studies encompassing a small number of DNA fragments, Malvaceae have been subdivided into nine subfamilies (i.e., Byttnerioideae, Grewioideae, Helicteroideae, Sterculioideae, Brownlowioideae, Dombeyoideae, Tilioideae, Bombacoideae, and Malvoideae), comprising two sister clades (i.e., Byttneriina and Malvadendrina) [10, 31]. Apart from the fact that Bombacoideae and Malvoideae together formed a well-supported clade named as Malvatheca, and Byttneriina (including Byttnerioideae and Grewioideae) formed a sister clade with the remaining Malvaceae taxa, relationships of other subfamilies have been poorly resolved [35–41]. Until recent studies based on plastid genomes, the phylogenetic relationships among nine subfamilies have been largely improved [42–46]. However, since the datasets in their analysis lack one to three subfamily members in most cases, the relationships among several of its nine subfamilies still remain unclear. Only Cvetković et al. [47] reported the complete phylogenetic tree based on cp genomes, which exhibited a well-supported topology confirming the split of the family into Byttneriina and Malvadendrina. Defectively, in their topology, the clade including Brownlowioideae, Dombeyoideae, and Tilioideae was supported as sister clade to Malvatheca with moderate bootstrap (bs = 72). Moreover, only one species belonging to Brownlowioideae was included in their dataset. Thus, further phylogenetic analysis including more species in Brownlowioideae to clarify the phylogeny within Malvaceae is necessary.

In Malvaceae, many studies have assessed the possibility that cp genomes can be used to clarify the phylogenetic relationships, or to improve topology of phylogenetic tree among its subfamilies [42–44, 46, 48]. Since the cp genome of *Gossypium hirsutum* representing the first plastome in the Malvaceae was reported in 2006 [49], complete cp genome of a large number of species in Malvaceae were sequenced. Up to Oct 21, 2022, a total of 296 records of complete cp genome were retrieved from Genbank, including 132 species from nine subfamilies, but only one complete cp genome of Brownlowioideae has been retrieved. Here, we newly sequenced and assembled the plastome of *Diplodiscus trichospermus* recognized in Brownlowioideae and downloaded the allied genomes from public database to construct the dataset representing all subfamilies of Malvaceae, aiming to greatly clarify the phylogeny of Malvaceae, especially the phylogenetic position of Brownlowioideae.

## Results

### Plastome structure and RSCU of *Diplodiscus trichosperma*

**Fig. 1:**
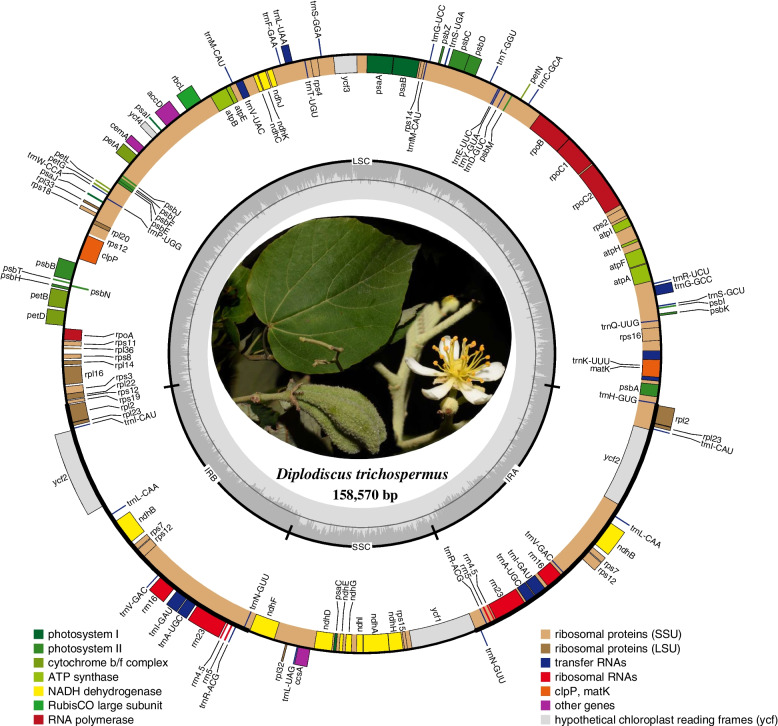
Plastid genome map of *Diplodiscus trichosperma*. Genes inside the circle are transcribed clockwise, genes outside are transcribed counterclockwise. Genes are color-coded to indicate functional groups. The circle inside the GC content graph marks the 50% threshold

The complete cp genome of *Diplodiscus trichosperma* was successfully assembled and annotated (Fig. 1). Like the most species of Malvaceae, its plastome has a typical quadripartite structure [44, 50, 51], namely, the two repeat regions (IRs) are separated by a large single copy region (LSC) and a small single copy region (SSC). The total length was 158,570 bp with 37.2% GC content. 112 unique genes were found in complete cp genome of *Diplodiscus trichosperma*, including 78 protein-coding genes, 30 tRNA genes, 4 rRNA genes. LSC (87,808 bp), SSC (19,558 bp), and IR (25,602 bp) included 82, 13, and 17 unique genes respectively.

**Fig. 2:**
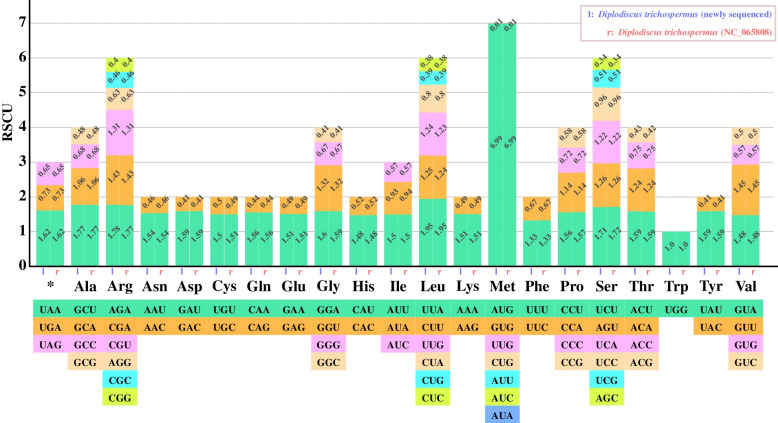
The RSCU values of the 20 amino acids and one stop codon for the *Diplodiscus trichosperma* cp genome. Bar color corresponds to the codon for each amino acid

Codon usage bias, preferential or non-random use for synonymous codons, is a universal phenomenon observed in organisms [52–54]. It is generally affected by gene mutation, natural selection, and genetic drift [55, 56]. To analyze the frequency of codon usage, a total of 78 unique coding sequences were extracted from cp genome to calculate the relative synonymous codon usage values (RSCU). All genes began with the codon AUG, except *ndh*D gene with the non-AUG start codon (GUG) (Fig. 2). 31 codons in the 78 unique CDSs have a positive bias (RSCU value > 1), where 29 are A- or U-ending codons, and the most frequent was AUU isoleucine-encoding (987 occurrences). Correspondingly, the negative bias (RSCU value < 1) was found in 33 codons where 30 are G- or C-ending, and the least frequent codon was GUG methionine-encoding (only 1 occurrence). All stop codons were found, especially the UAA with 42 occurrences showed the strong codon usage bias (RSCU value = 1.62).

### Phylogenetic analysis

**Fig. 3:**
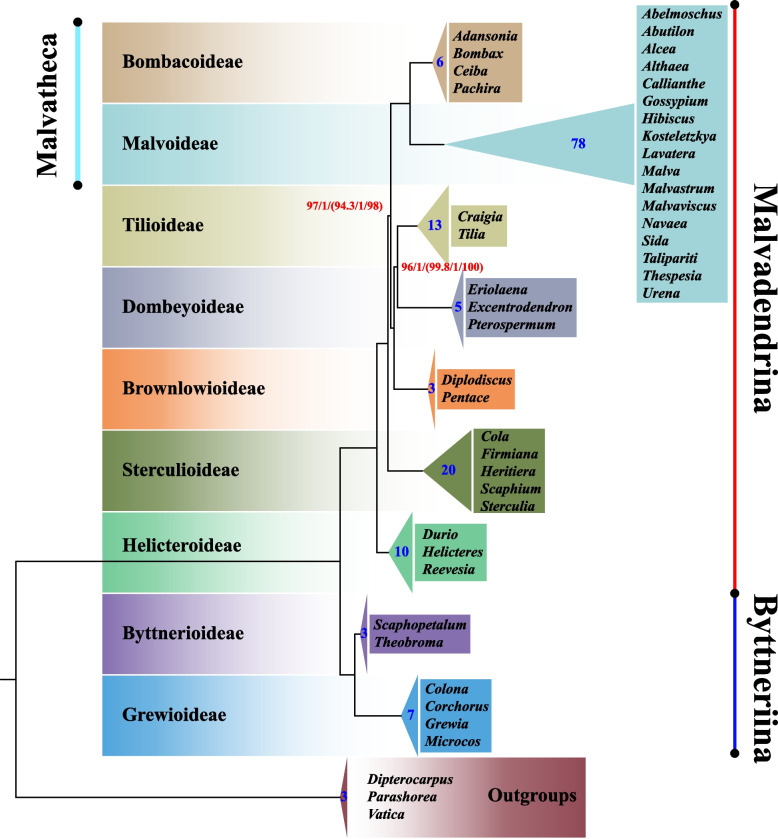
The plastid phylogeny of the Malvaceae inferred from complete cp genome sequences. The numbers at each node indicate the bootstrap support (BS) / posterior probability (PP) / (SH-aLRT support / aBayes support / ultrafast bootstrap support). The unlabeled nodes indicate 100% / 1.0 / (100% / 1 / 100%) support values. Clades are color-coded according to subfamily. The blue numbers deposited in triangle shows the species number in each subfamily

To reveal the phylogenetic position of Brownlowioideae in Malvaceae, we reconstructed the Bayesian inference (BI) and maximum likelihood (ML) trees based on 148 genomes (including all genes and intergenic spacers) covering all subfamilies in Malvaceae and three outgroups. All 148 cp genome sequences have a typical quadripartite structure, and its genome size ranges from 157,936 bp to 168,953 bp. The IR length ranges from 23,726 bp to 34,496 bp. By aligning the genome to a reference sequence, we found that SSC in almost half of the 148 sequences have forward read orientation (the SSC orientation of *Malva wigandii* was designed as a reference), while the remains possess the reverse orientation. Thus, we normalized the orientation of all sequences according to LSC-IRb-SSC-IRa for subsequent phylogenetic analyses. The analysis recovered a robust phylogenetic backbone of Malvaceae, and a closer relationship between Brownlowioideae, Tilioideae, and Dombeyoideae (Fig. 3 and Additional file 1). All phylogenetic trees constructed by RAxML, IQ-TREE 2 or MrBayes have strong support values in each node of their topology, and the inferred relationships are completely congruent among these trees (Additional file 1).

**Fig. 4:**
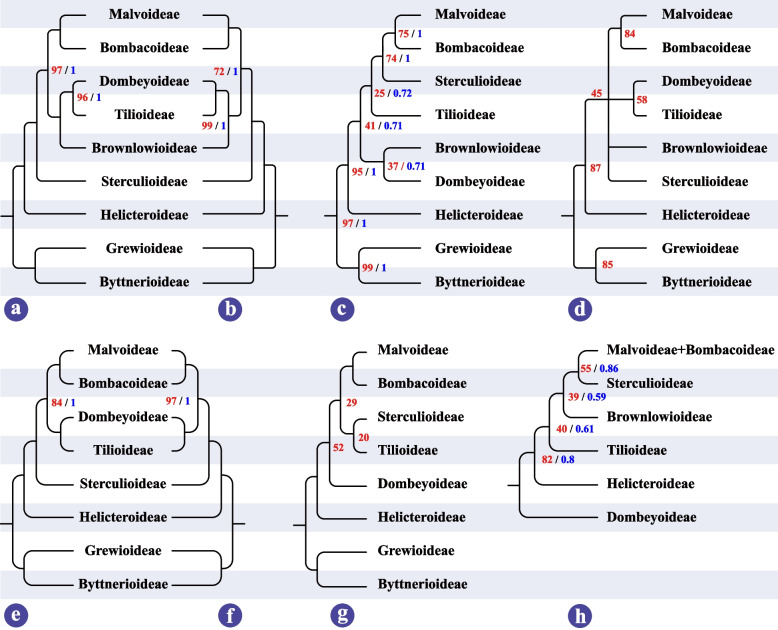
Phylogenetic relationships among subfamilies of Malvaceae. The number labeled by red and blue font indicates the ML bootstrap values and BI posterior probability respectively. The unlabeled nodes indicate 100% and (or) 1.0 support values. **a** In this study. **b** From Cvetković et al. [47]. **c** From Hernández-Gutiérrez and Magallón [41]. **d** From Alverson et al. [31]. **e** From Wang et al. [46]. **f** From Li et al. [45]. **g** From Conover et al. [42]. **h** From Nyffeler et al. [37]

Unsurprisingly, Grewioideae and Byttnerioideae formed a clade named as Byttneriina, which was a sister to the clade comprising the residual subfamilies in Malvaceae named as Malvadendrina. In the Malvadendrina clade, Malvoideae and Bombacoideae formed a close sister clade named as Malvatheca; subfamily Helicteroideae occupied the most basal position and was followed by Sterculioideae which was sister to the alliance of Malvoideae, Bombacoideae, Dombeyoideae, Tilioideae, and Brownlowioideae; Brownlowioideae together with the clade including Dombeyoideae and Tilioideae formed a sister clade to Malvatheca. Overall, the topology we recovered is basically identical to previous analysis based on the cp genomes, except for some results based on molecular fragments where the topology of the phylogenetic tree has weak support, and the phylogenetic position of some subfamilies such as Sterculioideae and Dombeyoideae is unstable (Fig. 4).

Tree topology tests were performed on the phylogenetic trees previously considered controversial (Fig. 4). The statistical tests rejected hypotheses b (Brownlowioideae and Dombeyoideae formed a sister group and Sterculioideae was close to Malvatheca), c (Sterculioideae and Tilioideae formed a close clade which was sister to Malvatheca) and d (Dombeyoideae formed the earliest divergent clade), but failed to reject hypothesis a (Helicteroideae located at the most basal position and Brownlowioideae formed a sister to the clade comprising Tilioideae and Dombeyoideae) which possess high confidence (Table 1). The result indicated a reliable phylogenetic tree of Malvaceae where the systematic position of Brownlowioideae was resolved with great certainty.

**Table 1:** Statistical tests of alternative tree topology hypotheses conducted by IQ-TREE 2

Tree	logL	deltaL	bp-RELL	p-KH	p-SH	p-WKH	p-WSH	c-ELW	p-AU
a	-1,014,060.351	0	1 +	1 +	1 +	1 +	1 +	1 +	1 +
b	-1,014,228.252	167.9	0-	0-	0.0001-	0-	0-	1.37e-25-	2.29e-05-
c	-1,014,230.306	169.95	0-	0-	0.0001-	0-	0-	1.16e-24-	1.06e-82-
d	-1,014,632.753	572.4	0-	0-	0-	0-	0-	4.61e-159-	0.00018-

a Helicteroideae located at the most basal position and Brownlowioideae formed a sister to the clade comprising the Tilioideae and Dombeyoideae (present study); b Brownlowioideae and Dombeyoideae formed a sister group and Sterculioideae was close to Malvatheca (structurally identical to Hernández-Gutiérrez and Magallón [41]); c Sterculioideae and Tilioideae formed a close clade which was sister to Malvatheca (structurally identical to Conover et al. [42]); d Dombeyoideae formed the earliest divergent clade (structurally identical to Nyffeler et al. [37]). a, b, c, and d corresponding to Additional file 4a, b, c, and d respectively. The value of deltaL indicates that logL differs from the maximal log1 in the comparison. bp-RELL, bootstrap proportion using RELL method [57]; p-KH, *p*-value of one-sided Kishino-Hasegawa test [58]; p-SH, *p*-value of Shimodaira-Hasegawa test [59]; c-ELW, Expected Likelihood Weight [60]. p-AU, *p*-value of approximately unbiased (AU) test [61]. Plus signs denote the 95% confidence sets. Minus signs denote significant exclusion. All test performed on 10,000 replicates using the RELL method

### Comparison of genome structures

**Fig. 5:**
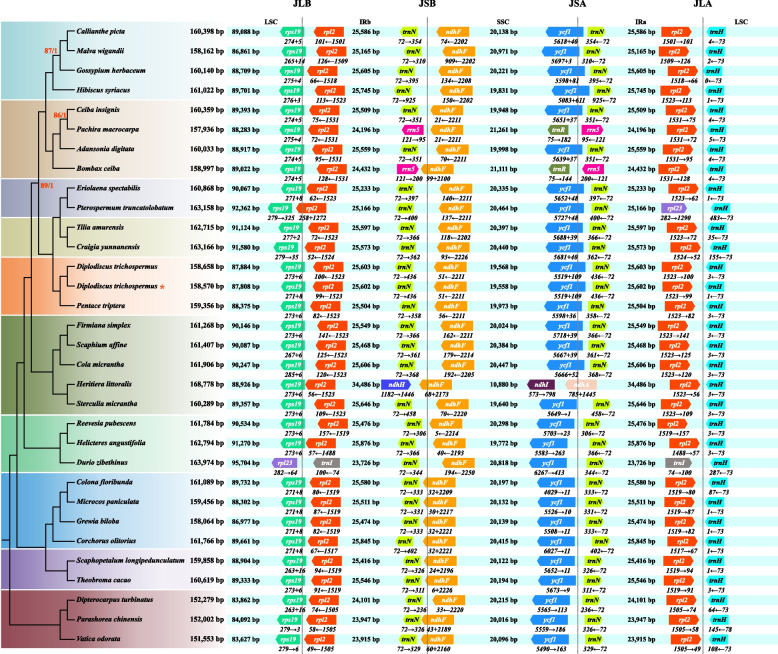
Comparison of the IR-boundaries among species of Malvaceae. The number at the tail and tip of mini arrows showed the gene length and bp distance from the corresponding junction site respectively. The sum of the numbers at both ends of the ‘ + ’ is the gene length, and each number showed the bp distance from the corresponding junction site. In the phylogenetic tree on the left, clades are color-coded according to subfamily. The ML bootstrap values / BI posterior probability was marked at each node. The unlabeled nodes indicate 100% / 1.0 support values. The sequence newly generated in this study was marked by asterisk

**Fig. 6:**
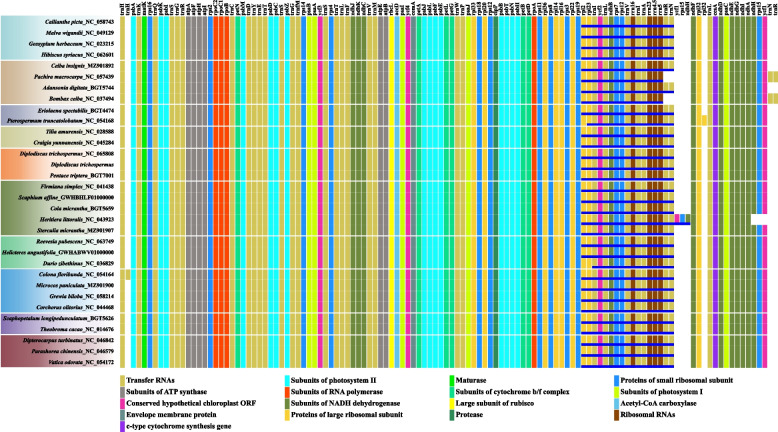
The gene order map of Malvaceae cp genomes. the genes marked with a blue underline are located in IRb region. The IRa region was removed from analysis

To analyse the junctions of four distinct regions (LSC, IRb, SSL, and IRa) in cp genomes within Malvaceae, a dataset including the sequence newly generated in this study and another 31 species downloaded from public database was constructed. This dataset comprised all subfamily members of Malvaceae and three outgroups. We visualized the IR boundaries and the gene order of Malvaceae showed as Figs. 5 and 6 respectively. The contraction and expansion of IRs exhibited similar patterns (Fig. 5), namely, *rps*19 and *rpl*2 located in the vicinity of the LSC/IRb junctions (JLB); *trn*N and *ndh*F located in IRb/SSC (JSB); *ycf*1 and *trn*N located in SSC/IRa (JSA); *rpl*2 and *trn*H located in IRa/LSC (JLA). Apparently, the genes enclosing each junction site or bp distance of the genes from the junction site are not coincident with the phylogenetic tree. In subfamily Bombacoideae, although *Ceiba insignis* and *Pachira macrocarpa* showed a close relationship, their junction genes were different. In Byttnerioideae and Grewioideae, their junction patterns were completely identical, namely *rps*19 extended from LSC to IRb; the whole *rpl*2 and *trn*N were included in IRs; *ndh*F mostly existed in SSC and partially in IRb region; *ycf*1 completely located in SSC; unsurprisingly, the *trn*H gene of each cp genome presented completely in LSC region. The similar structural feature was also observed in subfamily Bombacoideae and Malvoideae, except that *ndh*F was mostly localized in SSC and *ycf*1 started from the IRa region and integrated into the SSC region. In conclusion, the junction patterns of most Malvaceae species were similar to Myrtaceae [62], Lythraceae [63], Combretaceae [64] and Brassicaceae [65] in Malvids, namely *rps*19 and *rpl*2 in JLB, *trn*N and *ndh*F in JSB, *ycf*1 and *trn*N in JSA, *rpl*2 and *trn*H in JLA.

In addition, the gene order of cp genome is highly conserved among subfamilies in Malvaceae (Fig. 6), except for partial gene order changes caused by the contraction or expansion in the IR regions or gene duplications in single-copy regions. The contraction of IR regions in *Pachira macrocarpa* and *Bombax ceiba* led to the transfer of *trn*N and *trn*R to SSC region, while the contraction of IR regions in *Durio zibethinus* and *Pterospermum truncatolobatum* resulted in the transfer of *rpl*2 and *rpl*2 together with *rpl*23 to LSC respectively. The expansion of the IR regions in *Heritiera littoralis* led to the loss of *ycf*1, *rps*15, and *ndh*H from SSC. Additionally, the duplications of *trn*H in LSC and *rpl*32 in SSC were observed in *Colona floribunda* and *Pterospermum truncatolobatum* respectively.

### SSRs, palindromic sequences and conserved sequence analysis

**Fig. 7:**
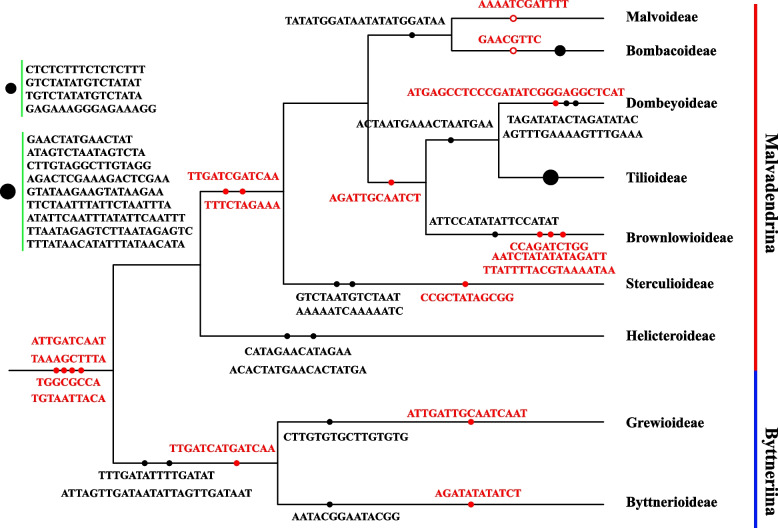
The SSRs and palindromic sequences shared within or among subfamilies. The black and red sequences indicate SSRs and palindromic sequences respectively. The solid circle indicates the presence of sequence while the hollow circle indicates the absence of sequence

Combining the sequences newly generated in this study and reported in public database, a total of 145 cp genomes, which covered 9 subfamilies, 42 genera, and 145 species (see Additional file 2 for detail), were used to investigate the sequence features. Almost of all subfamilies in Malvaceae have its specifical variable number of tandem repeat (VNTR) sequences, except Malvoideae where we not found complete conservation SSRs (Fig. 7). Only one conservation VNTR was observed in Grewioideae, Byttnerioideae, and Brownlowioideae respectively, while at least two sequences in other subfamilies, especially four and nine in Bombacoideae and Tilioideae respectively. The members in Malvatheca and in the clade comprising Dombeyoideae and Tilioideae shared the VNTR sequences "TATATGGATAATATATGGATAA" and "ACTAATGAAACTAATGAA" respectively. Byttnerioideae and Grewioideae were observed to share two conserved VNTR sequences located in the intergenic spacer between *ycf*4 gene and *cem*A gene. Furthermore, the palindrome sequences shared among subfamilies were also analysed. In Brownlowioideae, three palindrome sequences were observed in its members, two of which were situated in IRs and one in *trn*L-UAA gene. A palindrome sequence ("AGATTGCAATCT") which posited in *ndh*A gene was shared by Dombeyoideae, Tilioideae, and Brownlowioideae. The palindrome sequence ("CCGCTATAGCGG") in *rpo*B gene was observed as completely conservation in Sterculioideae. In Grewioideae, Byttnerioideae, and Dombeyoideae, one shared palindromic sequence was found in the intergenic spacer in each subfamily (Fig. 7). The clade comprising Sterculioideae, Brownlowioideae, Tilioideae, Dombeyoideae, and Malvatheca shared two palindromic sequences ("TTGATCGATCAA" and "TTTCTAGAAA") located in IRs. The clade Byttneriina shared "TTGATCATGATCAA". Interestingly, the palindromic sequence "AAAATCGATTTT" and "GAACGTTC" are lost in Malvoideae and Bombacoideae respectively. All Malvaceae members shared four palindromic sequences (Fig. 7). In addition, we found more conserved sequences in the branch nodes containing two or more closely related subfamilies (see Additional file 3 for detail). These sequences may provide evolutionary evidence for the divergence of each subfamily.

## Discussion

### Chloroplast genome of Malvaceae

**Fig. 8:**
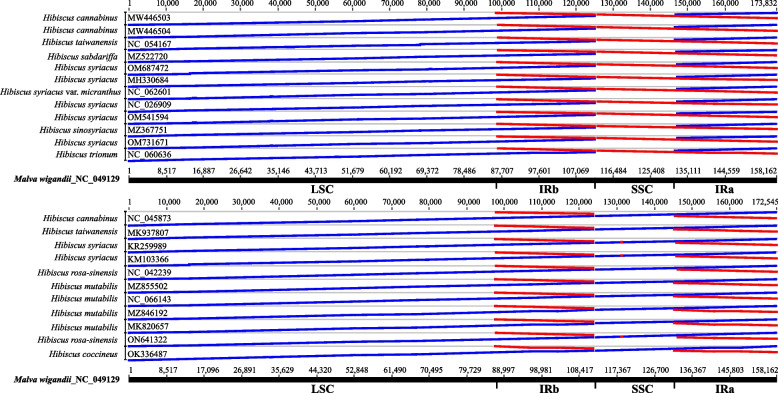
The orientation of cp genomes for *Hibiscus*. *Malva wigandii* (NC_049129) was designed as the target sequence. The blue line indicates the same direction as the target sequence, while the red line indicates the opposite direction

In this study, 145 representative taxa from 42 genera of Malvaceae and three outgroups were included in analysis dataset. These cp genomes have wide variations of SSC orientation as claimed by Cheng et al. [51], especially for genus *Hibiscus* as Fig. 8. However, whether this is the case or not, further third-generation sequencing data may be necessary to confirm it. There is no doubt that the inconsistency of the reference selection when annotating plastomes also can result in a variable read orientation [66].

The majority of cp genomes in angiosperm have conservative quadripartite structure, namely two inverted repeats were separated by one small single copy and one large single copy [67], and the genome size ranges from 11 kb [8] to 240 kb (in *Pelargonium transvaalense*, Accession: NC_031206.1). In our dataset, no exception was observed in the quadripartite cp genome structure of Malvaceae, and its genome size which ranged from 157,936 bp to 168,953 bp was also within the general size for angiosperms, suggesting that the species exhibited extremely conserved cp genome size and structure in Malvaceae.

The IR region is important to stabilize cp genome structure, and its slower nucleotide substitution rates compared with single-copy regions can enhance copy-correction activity [68]. About 10,000 bp variations of IR length of cp genome in Malvaceae indicated the noticeable genetic differences generally resulting from the contraction or expansion of the IR regions [69]. The changes in IR regions may result in a rearrangement of their gene order [70]. An obvious expansion in IRs of *Heritiera littoralis* resulted in that three genes (*ycf*1, *rps*15, and *ndh*H) general located in SSC were transferred to IR regions, while *Bombax ceiba* (Bombacoideae), *Pachira macrocarpa* (Bombacoideae), *Durio zibethinus* (Helicteroideae), and *Pterospermum truncatolobatum* (Dombeyoideae) have a contrary case where some genes general in IRs were transferred to sing-copy regions (Fig. 6). The events of IRs contraction or expansion also occurred in Rutaceae [71], Sapindaceae [72], Meliaceae [73], Onagraceae [74], and Thymelaeaceae, which all belong to the order Malvids. Especially in Thymelaeaceae, the IR length is nearly twice of it in most angiosperms, resulting in about 2–3 kb residue in SSC region which only contains the *ndh*F and *rpl*32 genes [75].

The gene duplication in cp genome is an essential source of organelle evolution, new genes, and new genetic functions [76]. The gene duplication in single-copy regions was usually caused by the expansion of the IR regions [76], and only a few not involving the IRs have been documented in cp genomes, such as *psb*Z in *Wolffia* [77], *trn*Q-UUG in *Epimedium* and Geraniaceae [78, 79], *psb*A and *trn*T-GGU in *Pinus* [80, 81], and *psb*J in *Trachelium* [82]. Gene duplication events not involving the IRs were detected in a few Malvaceae species (*trn*H occurs in LSC of *Colona floribunda*, and *rpl*32 occurs in SSC of *Pterospermum truncatolobatum*), indicating that the cp genome of Malvaceae may be undergoing an evolution of new genes or new gene functions to further adapt to the changeable environment.

Interestingly, the above genetic events (IR regions contraction and expansion, and gene duplication) in Malvaceae are not exclusive to a single subfamily or several closely related subfamilies, but are scattered into different subfamilies. Thus, it is evident that gene losses or gains in the repeat regions or gene duplication in single-copy regions may not indicate a phylogenetic signal at the subfamily level, which is similar to the claims of Jansen et al. [83].

### Phylogenetic relationships inference

Malvaceae, which provides food, beverage, timber and traditional medicine for humans, especially most important fiber crops, is an importantly economical plant family in rosids [84]. However, the intrafamilial phylogenetic relationships are currently controversial, which may be caused by two primary reasons. One is that no sample of Brownlowioideae was included in the dataset, which thereby led to the fact that its phylogenetic position was unknown [46]; the other is that the phylogenetic trees were generally reconstructed by using one or a few loci, which resulted in different topologies with relatively low supports [37].

The plastome, general conservation, uniparental inheritance, and less prone to recombination between homologous copies [5, 49], is an ideal model for studying gene evolution and phylogenetic relationships. Compared to a limited number of DNA fragments which provide relatively little genetic variation, the whole cp genome sequences contain more integrated and adequate genetic information, and were regarded as an effective tool to investigate the phylogenetic relationships and gene evolution [4, 7, 85]. To produce a high-supported tree and clarify their phylogeny, we employed the whole cp genome sequences to evaluate the phylogenetic relationship among subfamilies in Malvaceae. Maximum likelihood analysis and Bayesian inference recovered a strongly supported phylogenetic backbone of Malvaceae. Our newly generated phylogenetic tree (Fig. 4a) is structurally identical to the one recently reported by Cvetković, et al. [47] (Fig. 4b). There is a moderate support between Malvatheca and the clade comprising Tilioideae, Dombeyoideae, and Brownlowioideae for the phylogenetic tree recovered by Cvetković, et al., while strongly supported value (BS = 97, PP = 1) for ours. The phylogenetic tree of Malvaceae is distinctly divided into two major branches, namely Byttneriina and Malvadendrina formed a sister group without controversy [31, 41, 46]. In addition, the Byttneriina including Byttnerioideae and Grewioideae shared the largest number of conserved sequences, and these sequences can be up to 182 bp in length (see Additional file 3 for detail), which may result in a distinct divergence from Malvadendrina.

Within the Malvadendrina clade, the close relationship of the Malvoideae and Bombacoideae was firstly identified [31, 36] and had no controversy for a long time. The majority of studies on phylogeny of Malvaceae showed that Helicteroideae located at the base of Malvadendrina (Fig. 4b, c, d, e, f, g), while based on concatenation of *atp*B, *mat*K and *ndh*F or *atp*B, *trn*K-*mat*K, *ndh*F, *rbc*L and ITS showed that Dombeyoideae was the first divergent (Fig. 4h) [37, 86]. The topologies between Tilioideae, Brownlowioideae, and Sterculioideae have been largely incongruent and remain unresolved. Especially for Brownlowioideae, its phylogenetic position remains largely unknown for that no sample in Brownlowioideae was included in analysis dataset [42, 45, 46]. Alverson et al. [31] tried to recover the phylogeny of the "core Malvales" based on the *ndh*F sequences which was the first dataset including the sample of Brownlowioideae, but the relationship between Brownlowioideae, Sterculioideae, Malvatheca, and the clade comprising the Dombeyoideae and Tilioideae had not been resolved (Fig. 4d). The tree reconstructed by concatenation of *atp*B, *mat*K and *ndh*F revealed the Brownlowioideae as sister to the clade comprising Sterculioideae and Malvatheca with weak support (Fig. 4h) [37]. Furthermore, the close relationship between Brownlowioideae and Dombeyoideae was reported by Hernández-Gutiérrez and Magallón [41], but the bootstrap support value is extremely low (Fig. 4c). Up to 2021, a robust relationship between Brownlowioideae, Tilioideae, and Dombeyoideae was confirmed by Cvetković, et al. [47] based on the cp genome data where Brownlowioideae was represented by only one sequence, namely Brownlowioideae is sister group to the other two subfamilies. The identical phylogeny was also recovered by our dataset comprising three sequences in Brownlowioideae. In addition, the systematic position of Sterculioideae is generally controversial. Some studies argued it formed as a sister to Malvatheca clade (Fig. 4c, h) [37, 41], while others supported it as a close relative to Tilioideae (Fig. 4g) [42]. Recently, cp genomic data resolved Sterculioideae as the base divergent clades after Helicteroideae in Malvadendrina [45–47], which is consistent with our study.

## Conclusions

Clarifying the phylogenetic backbone of Malvaceae may contribute to exploiting the alternative food, drink, fiber, and wood resources from this economically important family and protect them better in the future. Here, we recovered a robust phylogenetic tree of "core Malvales", and revealed Brownlowioideae was sister group to Tilioideae and Dombeyoideae. The result exhibited that the cp genomic data not only can improve resolution of phylogenetic relationship among orders, families or even more genera, but also can resolve the phylogeny perfectly at subfamily level. Despite robust support values in every internode among subfamilies, more morphological synapomorphies are still required to support this phylogenetic relationship derived from cp genomes. In addition, the analysis of this study showed that the expansion or contraction of IR regions and gene duplication in single-copy regions are scattered in different subfamilies, so that they may not provide obvious phylogenetic signals at the subfamily level.

## Materials and methods

### Plant materials and total DNA extraction

Plant samples of *Diplodiscus trichosperma* were collected from Jianfeng Town, Ledong Li Autonomous County, Hainan Province, China (N 18.7001767, E 108.7062028). The voucher specimen (Mingsong Wu, WuMS216) was deposited in the herbarium of Sichuan University (SZ).

Total genomic DNA was extracted from young developing leaf tissues collected from the living plant, and dried immediately by silica gel using the modified CTAB method [87]. Genome skimming was conducted by Novogene Bioinformatics Technology Co. Ltd. (Tianjin, China) using next-generation sequencing technologies on the Illumina NovaSeq 6000 platform with 150 bp paired-end reads and 350 bp insert size.

### Genome assembly and annotation

A total of 3.21 Gb paired-end sequencing data was generated to proceed the further analysis. The GetOrganelle pipeline [88], Bandage [89] and Plastid Genome Annotator [90] were employed to assemble the complete plastome, visualize the assemblies and annotate the genome features respectively. The cp genome of *Malva wigandii* (NC_049129) was designated as a reference for annotation. The start/stop codons, intron/exon boundaries, and tRNA genes for the preliminary annotation result were manually adjusted by Geneious Prime 2020.1.2 (Biomatters Ltd., Auckland, New Zealand).

### Relative synonymous codon usage and gene map

The relative synonymous codon usage (RSCU) of protein-coding genes was calculated and visualized using a python script written by Mingsong Wu. The circle gene maps of the plastid genes were drawn by OGDRAW [91].

### Comparative analysis of genome structure

To compare the cp genome structural features of all subfamilies in Malvaceae, we downloaded all available cp genomes of Malvaceae from Genbank (https://www.ncbi.nlm.nih.gov/nuccore/), CGIR (https://ngdc.cncb.ac.cn/cgir/), and reported by Cvetković et al. [47]. A typical species was selected for each genus, with the exception of the subfamily Malvoideae, where only 4 species were selected to represent 4 of the 16 genera. A total of 32 cp genomes were employed as the analysis dataset, covering all subfamily members in the Malvaceae and three outgroups. In addition, we reannotated all sequences using the plastome of *Malva wigandii* (NC_049129) as a reference. The LasterZ plugin in Geneious was used to normalize the orientation of all sequences according to LSC-IRb-SSC-IRa. The genome sequences were aligned by MAFFT v.7.308 [92] with default parameters. The gene order and gene content adjacent to the borders of the two single copies were visualized and compared by a python script written by Mingsong Wu.

### SSRs, palindromic sequences and completely conserved sequences identification

Simple sequence repeats (SSRs) grouped into four categories (i.e., P-SSRs, C-SSRs, I-SSRs, and VNTRs) were identified and localized using Krait software [93]. The default parameters were set for all SSRs analysis in this study. Palindromic sequences finder in NovoPro online tools (https://www.novoprolabs.com/tools/dna-palindrome) was used to find the palindromic sequences. The completely conserved sequences within or among the subfamilies were identified using a python script written by Mingsong Wu.

### Phylogenetic inference and tree topology comparison

We employed *Pentace triptera* together with *Diplodiscus trichosperma* to represent the subfamily Brownlowioideae, and combined 142 allied genomes downloaded from public database to reconstruct the phylogenetic backbone of Malvaceae, and clarify the phylogenetic position of subfamily Brownlowioideae within "core Malves". A total of 148 genomes (see Additional file 2 for detail) were included in this dataset, and all sequences were reannotated with the reference genome. The orientation of all sequences was standardized according to LSC-IRb-SSC-IRa. Whole cp genome sequences were used to construct the data matrix and MAFFT v.7.308 [92] was employed to align the data matrix. The maximum likelihood (ML) and Bayesian inference (BI) phylogenetic trees were reconstructed using RAxML [94] and MrBayes [95] on CIPRES cluster (https://www.phylo.org/) respectively. The parameters for ML were GTRGAMMA substitution model and 1000 bootstraps, and for BI were as follows: lset nst = 6; rates = gamma; mcmcp ngen = 1000000; relburnin = yes; burninfrac = 0.25; printfreq = 1000; samplefreq = 1000; nchains = 4; savebrlens = yes; other settings = default. IQ-TREE 2 [96] was employed to infer another ML tree and performed the SH-aLRT test, aBayes test, as well as ultrafast bootstrap test with 10,000 replicates. The analyses were run with the command "iqtree2 -s inputfile.phy -m MFP –abayes –alrt 10000 -B 10000 -T AUTO".

The tree topologies were generated using TreeGraph2 [97] to reflect the controversial hypotheses on the phylogeny of subfamilies in Malvaceae. Four aspects of Malvadendrina phylogeny that have been previously considered controversial were tested under a likelihood theory framework (see Additional file 4 for detail): (a) Helicteroideae located at the most basal position and Brownlowioideae formed a sister to the clade comprising the Tilioideae and Dombeyoideae; (b) Brownlowioideae and Dombeyoideae formed a sister group and Sterculioideae was close to Malvatheca; (c) Sterculioideae and Tilioideae formed a close clade which was sister to Malvatheca; and (d) Dombeyoideae formed the earliest divergent clade. Bootstrap proportion (BP) test [57], Kishino-Hasegawa (KH) test [58], Shimodaira-Hasegawa (SH) test [59], approximately unbiased (AU) test [61], weighted KH (WKH), weighted SH (WSH) and expected likelihood weight (ELW) [60] were performed in IQ-TREE 2 [96]. The number of RELL replicates was specified as 10,000. Probability values (*p*-values) of the KH, SH and AU test smaller than 0.05 indicate that the hypothesis was rejected (marked with a—sign). The command was "iqtree2 -s inputfile.phy -z inputfile.trees -n 0 -zb 10000 -zw -au".

### Supplementary Information

**Additional file 1.** The phylogenetic trees of Malvaceae. Clades are color-coded according to subfamily. a, b and c indicate that ML tree recovered by RAxML, BI tree recovered by MrBayes and ML tree recovered by IQ-TREE 2 respectively. Numbers at each node in a and b indicate the BS and PP values respectively. Numbers at each node in c indicate SH-aLRT support/aBayes support/ultrafast bootstrap supports.

**Additional file 2.** The species in Malvaceae covered in dataset. The yellow background indicates the plastome of *Diplodiscus trichospermus* newly sequenced in this study. Species marked in red font are designated as outer groups.

**Additional file 3.** The conserved sequences shared among subfamilies. The blue color indicated the sequences located in the IRs. 

**Additional file 4.** The hypothetical tree topologies generated by TreeGraph 2. a Helicteroideae located at the most basal position and Brownlowioideae formed a sister to the clade comprising Tilioideae and Dombeyoideae (present study); b Brownlowioideae and Dombeyoideae formed a sister group and Sterculioideae was close to Malvatheca; c Sterculioideae and Tilioideae formed a close clade which was sister to Malvatheca; d Dombeyoideae formed the earliest divergent clade.

## Data Availability

The complete annotated sequence of *Diplodiscus trichospermus* is deposited in the NCBI database (https://www.ncbi.nlm.nih.gov/) (GenBank accession number: OP572286). The *D. trichospermus* material was obtained from Ledong, Hainan, China, and the specimen was subsequently deposited in the herbarium of Sichuan University (SZ). The other cp genomes used in this study were downloaded from the NCBI.
