# Supplementary material for: The complete chloroplast genome of Diplodiscus trichospermus and phylogenetic position of Brownlowioideae within Malvaceae
Source: BMC Genomics. 2023 Sep 26;24:571. doi: 10.1186/s12864-023-09680-z (PMC10521492; doi:10.1186/s12864-023-09680-z)
Supplement: Supplementary file 1 — Additional file 1. The phylogenetic trees of Malvaceae. Clades are color-coded according to subfamily. a, b and c indicate that ML tree recovered by RAxML, BI tree recovered by MrBayes and ML tree recovered by IQ-TREE 2 respectively. Numbers at each node in a and b indicate the BS and PP values respectively. Numbers at each node in c indicate SH-aLRT support/aBayes support/ultrafast bootstrap supports. [file 12864_2023_9680_MOESM1_ESM.pdf]

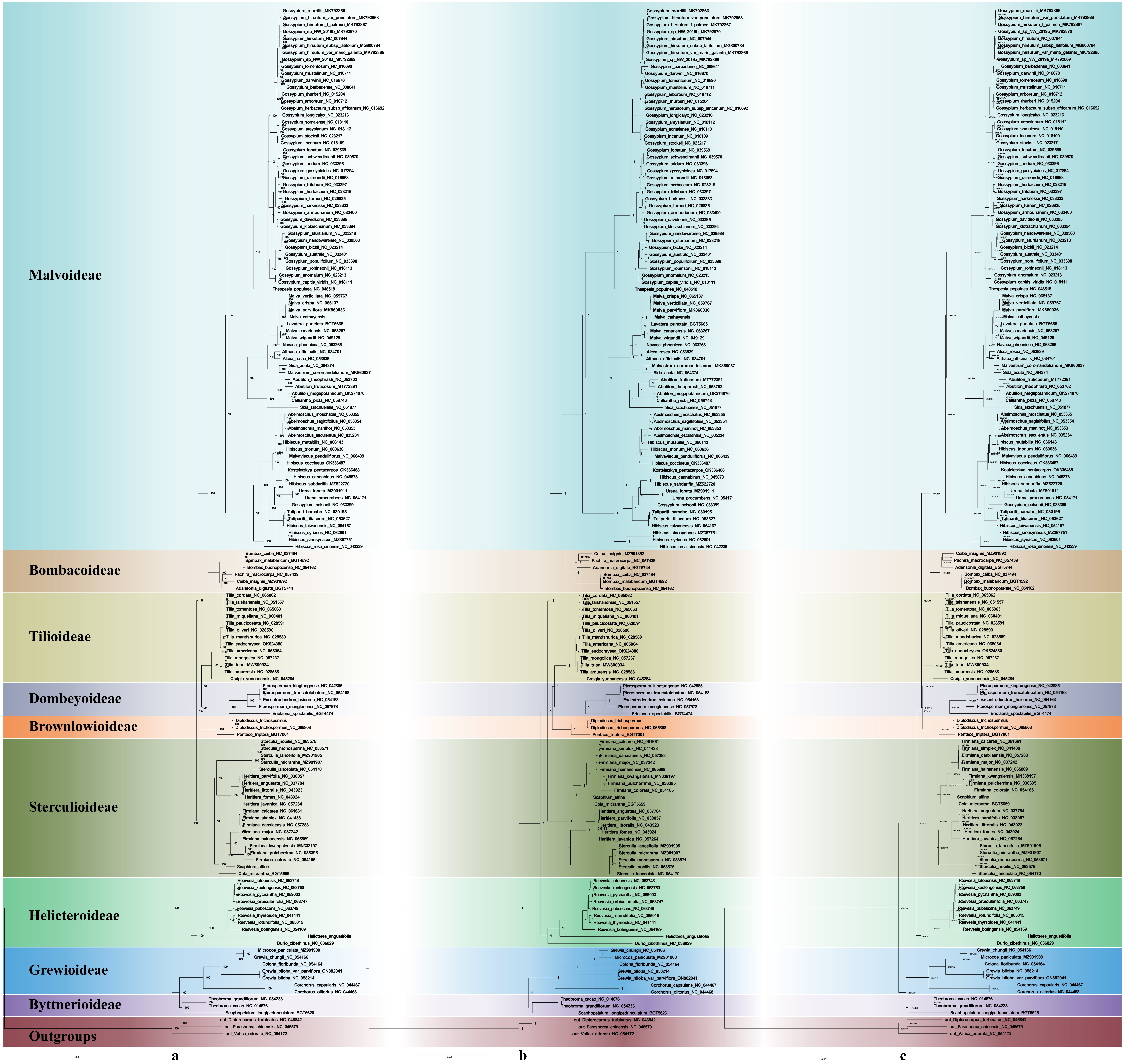

Additional file 1 The phylogenetic trees of Malvaceae. Clades are color-coded according to subfamily. a, b and c indicate that ML tree recovered by RAxML, BI tree recovered by MrBayes and ML tree recovered by IQ-TREE 2 respectively. Numbers at each node in a and b indicate the BS and PP values respectively. Numbers at each node in c indicate SH-aLRT support/aBayes support/ultrafast bootstrap supports
