# Supplementary material for: The complete chloroplast genome of Diplodiscus trichospermus and phylogenetic position of Brownlowioideae within Malvaceae
Source: BMC Genomics. 2023 Sep 26;24:571. doi: 10.1186/s12864-023-09680-z (PMC10521492; doi:10.1186/s12864-023-09680-z)
Supplement: Supplementary file 2 — Additional file 2. The species in Malvaceae covered in dataset. The yellow background indicates the plastome of Diplodiscus trichospermus newly sequenced in this study. Species marked in red font are designated as outer groups. [file 12864_2023_9680_MOESM2_ESM.pdf]

Additional file 2 The species in Malvaceae covered in dataset. The yellow background indicates the plastome of *Diplodiscus trichospermus* newly sequenced in this study. Species marked in red font are designated as outer groups.

seq_name	Sequence_ID	Source	subfamily	length	LSC_length	IR_length	SSC_length	GC
Adansonia digitata	BGT5744	Cvetkovic et al. 2021	Bombacoideae	160033	88917	25559	19998	36.87927
Bombax buonopozense	NC_054162	Genbank	Bombacoideae	160876	89347	25646	20237	36.84826
Bombax ceiba	NC_037494	Genbank	Bombacoideae	158997	89022	24432	21111	36.80887
Bombax malabaricum	BGT4592	Cvetkovic et al. 2021	Bombacoideae	159004	89029	24432	21111	36.80536
Ceiba insignis	MZ901892	Genbank	Bombacoideae	160359	89393	25509	19948	36.84109
Pachira macrocarpa	NC_057439	Genbank	Bombacoideae	157936	88283	24196	21261	36.90989
Diplodiscus trichospermus	OP572286	Genbank	Brownlowioideae	158570	87808	25602	19558	37.17033
Diplodiscus trichospermus	NC_065808	Genbank	Brownlowioideae	158658	87884	25603	19568	37.14972
Pentace triptera	BGT7001	Cvetkovic et al. 2021	Brownlowioideae	159356	88375	25504	19973	37.07736
Scaphopetalum longipedunculatum	BGT5626	Cvetkovic et al. 2021	Byttnerioideae	159858	88904	25416	20122	37.05539
Theobroma cacao	NC_014676	Genbank	Byttnerioideae	160619	89333	25546	20194	36.87422
Theobroma grandiflorum	NC_054233	Genbank	Byttnerioideae	160606	89429	25496	20185	36.88032
Eriolaena spectabilis	BGT4474	Cvetkovic et al. 2021	Dombeyoideae	160868	90067	25233	20335	36.6636
Excentrodendron hsienmu	NC_054163	Genbank	Dombeyoideae	161276	89442	25703	20428	36.71904
Pterospermum kingtungense	NC_042885	Genbank	Dombeyoideae	162929	91535	25465	20464	36.39131
Pterospermum menglunense	NC_057978	Genbank	Dombeyoideae	162421	90754	25572	20523	36.50267
Pterospermum truncatolobatum	NC_054168	Genbank	Dombeyoideae	163158	92362	25166	20464	36.38191
Colona floribunda	NC_054164	Genbank	Grewioideae	161089	89732	25580	20197	37.29367
Corchorus capsularis	NC_044467	Genbank	Grewioideae	161088	88615	26063	20347	36.93695
Corchorus olitorius	NC_044468	Genbank	Grewioideae	161766	89661	25845	20415	36.76112
Grewia biloba	NC_058214	Genbank	Grewioideae	158064	86977	25474	20139	37.39751
Grewia biloba var parviflora	ON882041	Genbank	Grewioideae	158043	86957	25474	20138	37.40248
Grewia chungii	NC_054166	Genbank	Grewioideae	159861	88683	25519	20140	37.22109
Microcos paniculata	MZ901900	Genbank	Grewioideae	159456	88302	25511	20132	37.32817
Durio zibethinus	NC_036829	Genbank	Helicteroideae	163974	95704	23726	20818	35.82641
Helicteres angustifolia	GWHABWV01000000	CGIR	Helicteroideae	162794	91270	25876	19772	36.72064
Reevesia botingensis	NC_054169	Genbank	Helicteroideae	160128	88864	25499	20266	37.01851
Reevesia lofouensis	NC_063748	Genbank	Helicteroideae	161894	90624	25478	20314	36.80186
Reevesia orbicularifolia	NC_063747	Genbank	Helicteroideae	161764	90518	25469	20308	36.82402
Reevesia pubescens	NC_063749	Genbank	Helicteroideae	161784	90534	25476	20298	36.83306
Reevesia pycnantha	NC_059003	Genbank	Helicteroideae	161964	90657	25496	20315	36.78842
Reevesia rotundifolia	NC_065015	Genbank	Helicteroideae	161905	90689	25485	20246	36.82221
Reevesia thyrsoidea	NC_041441	Genbank	Helicteroideae	161786	90565	25466	20289	36.83755
Reevesia xuefengensis	NC_063750	Genbank	Helicteroideae	161859	90576	25487	20309	36.8092
Abelmoschus esculentus	NC_035234	Genbank	Malvoideae	163121	88071	28009	19032	36.73653
Abelmoschus manihot	NC_053353	Genbank	Malvoideae	163428	88194	28150	18934	36.70485
Abelmoschus moschatus	NC_053355	Genbank	Malvoideae	163430	88243	28128	18931	36.70807
Abelmoschus sagittifolius	NC_053354	Genbank	Malvoideae	163453	88314	28162	18815	36.68761
Abutilon fruticosum	MT772391	Genbank	Malvoideae	160357	89034	25646	20031	36.95879
Abutilon megapotamicum	OK274070	Genbank	Malvoideae	159256	87836	25602	20216	37.1402
Abutilon theophrasti	NC_053702	Genbank	Malvoideae	160446	89089	25604	20149	36.88655
Alcea rosea	NC_053839	Genbank	Malvoideae	160189	88046	25544	21055	37.00566
Althaea officinalis	NC_034701	Genbank	Malvoideae	159987	87878	25526	21057	36.99863
Callianthe picta	NC_058743	Genbank	Malvoideae	160398	89088	25586	20138	36.99173
Gossypium anomalum	NC_023213	Genbank	Malvoideae	159508	88103	25603	20199	37.32477
Gossypium arboreum	NC_016712	Genbank	Malvoideae	160230	88722	25617	20274	37.22961
Gossypium areysianum	NC_018112	Genbank	Malvoideae	159572	88182	25569	20252	37.36871
Gossypium aridum	NC_033396	Genbank	Malvoideae	160257	88720	25647	20243	37.26452
Gossypium armourianum	NC_033400	Genbank	Malvoideae	160068	88658	25593	20224	37.2904
Gossypium australe	NC_033401	Genbank	Malvoideae	159578	88223	25567	20221	37.15612
Gossypium barbadense	NC_008641	Genbank	Malvoideae	160317	88841	25591	20294	37.22936
Gossypium bickii	NC_023214	Genbank	Malvoideae	159422	88073	25583	20183	37.19687
Gossypium capitis-viridis	NC_018111	Genbank	Malvoideae	159467	88065	25602	20198	37.32057
Gossypium darwinii	NC_016670	Genbank	Malvoideae	160378	88906	25603	20266	37.23391
Gossypium davidsonii	NC_033395	Genbank	Malvoideae	160072	88628	25602	20240	37.31009
Gossypium gossypioides	NC_017894	Genbank	Malvoideae	159959	88777	25589	20004	37.31206
Gossypium harknessii	NC_033333	Genbank	Malvoideae	160129	88710	25599	20221	37.2968
Gossypium herbaceum	NC_023215	Genbank	Malvoideae	160140	88709	25605	20221	37.30798
Gossypium herbaceum subsp africanum	NC_016692	Genbank	Malvoideae	160315	88790	25620	20285	37.21798
Gossypium hirsutum f palmeri	MK792867	Genbank	Malvoideae	160418	88941	25593	20291	37.2265
Gossypium hirsutum	NC_007944	Genbank	Malvoideae	160301	88817	25602	20280	37.24493
Gossypium hirsutum subsp latifolium	MG800784	Genbank	Malvoideae	160347	90064	24998	20287	37.2355
Gossypium hirsutum var marie-galante	MK792865	Genbank	Malvoideae	160310	88819	25606	20279	37.24908
Gossypium hirsutum var punctatum	MK792868	Genbank	Malvoideae	160427	88948	25594	20291	37.23438
Gossypium incanum	NC_018109	Genbank	Malvoideae	159205	87879	25565	20196	37.39204
Gossypium klotzschianum	NC_033394	Genbank	Malvoideae	160097	88654	25604	20235	37.30363
Gossypium lobatum	NC_039569	Genbank	Malvoideae	160205	88811	25550	20294	37.27412
Gossypium longicalyx	NC_023216	Genbank	Malvoideae	160241	88667	24548	22478	37.24203
Gossypium morrillii	MK792866	Genbank	Malvoideae	160322	88845	25593	20291	37.23382
Gossypium mustelinum	NC_016711	Genbank	Malvoideae	160313	88824	25610	20269	37.22094
Gossypium nandewarense	NC_039568	Genbank	Malvoideae	159677	88278	25579	20241	37.13121
Gossypium nelsonii	NC_033399	Genbank	Malvoideae	162316	89760	26596	19364	36.80105
Gossypium populifolium	NC_033398	Genbank	Malvoideae	159444	88197	25577	20093	37.19613
Gossypium raimondii	NC_016668	Genbank	Malvoideae	160161	88654	25651	20205	37.30746
Gossypium robinsonii	NC_018113	Genbank	Malvoideae	159849	88486	25580	20203	37.16007
Gossypium schwendimanii	NC_039570	Genbank	Malvoideae	160199	88779	25551	20318	37.26677
Gossypium somalense	NC_018110	Genbank	Malvoideae	159539	88150	25569	20251	37.36516
Gossypium sp	MK792869	Genbank	Malvoideae	160456	88932	25619	20286	37.22952
Gossypium sp	MK792869	Genbank	Malvoideae	160464	88946	25602	20314	37.22953
Gossypium stocksii	NC_023217	Genbank	Malvoideae	159039	87886	25487	20179	37.41284
Gossypium sturtianum	NC_023218	Genbank	Malvoideae	159627	88251	25580	20216	37.13344

Gossypium thurberi	NC_015204	Genbank	Malvoideae	160264	88737	25628	20271	37.21797
Gossypium tomentosum	NC_016690	Genbank	Malvoideae	160433	88932	25615	20271	37.20432
Gossypium trilobum	NC_033397	Genbank	Malvoideae	160109	88668	25604	20233	37.29022
Gossypium turneri	NC_026835	Genbank	Malvoideae	159926	88889	25411	20215	37.26161
Hibiscus cannabinus	NC_045873	Genbank	Malvoideae	162903	90351	26533	19486	36.64942
Hibiscus coccineus	OK336487	Genbank	Malvoideae	160280	89121	26243	18673	36.92476
Hibiscus mutabilis	NC_066143	Genbank	Malvoideae	160880	89355	26300	18925	36.91944
Hibiscus rosa-sinensis	NC_042239	Genbank	Malvoideae	160951	89509	25598	20246	36.99387
Hibiscus sabdariffa	MZ522720	Genbank	Malvoideae	162428	90327	26242	19617	36.73874
Hibiscus sinosyriacus	MZ367751	Genbank	Malvoideae	160892	89747	25742	19661	36.84521
Hibiscus syriacus	NC_062601	Genbank	Malvoideae	161022	89701	25745	19831	36.82851
Hibiscus taiwanensis	NC_054167	Genbank	Malvoideae	162800	90175	26617	19391	36.836
Hibiscus trionum	NC_060636	Genbank	Malvoideae	160530	89272	26152	18954	36.89902
Kosteletzkya pentacarpos	OK336488	Genbank	Malvoideae	161777	90019	26390	18978	36.79942
Lavatera punctata	BGT5665	Cvetkovic et al. 2021	Malvoideae	158571	87145	25156	21114	37.09253
Malvastrum coromandelianum	MK860037	Genbank	Malvoideae	159872	88106	25506	20754	37.10719
Malvaviscus penduliflorus	NC_066439	Genbank	Malvoideae	160332	88722	26327	18956	36.96268
Malva canariensis	NC_063267	Genbank	Malvoideae	158437	87150	25170	20947	37.05006
Malva cathayensis	GWHAZNZ01000000	CGIR	Malvoideae	158794	87216	25406	20766	37.12294
Malva crispa	NC_065137	Genbank	Malvoideae	158400	87077	25107	21109	37.11932
Malva parviflora	MK860036	Genbank	Malvoideae	158412	87086	25107	21112	37.1184
Malva verticillata	NC_059767	Genbank	Malvoideae	158408	87085	25107	21109	37.11744
Malva wigandii	NC_049129	Genbank	Malvoideae	158162	86861	25165	20971	37.08919
Navaea phoenicea	NC_063266	Genbank	Malvoideae	158598	87182	25157	21102	37.08685
Sida acuta	NC_064374	Genbank	Malvoideae	159147	87171	25536	20904	37.25361
Sida szechuensis	NC_051877	Genbank	Malvoideae	159878	89426	25288	19876	36.88312
Talipariti hamabo	NC_030195	Genbank	Malvoideae	161729	89217	26471	19570	36.89876
Talipariti tiliaceum	NC_053627	Genbank	Malvoideae	161748	89190	26471	19616	36.87712
Thespesia populnea	NC_048518	Genbank	Malvoideae	160451	88981	25582	20306	37.2419
Urena lobata	MZ901911	Genbank	Malvoideae	162189	89633	26631	19294	36.71889
Urena procumbens	NC_054171	Genbank	Malvoideae	163336	90475	26635	19591	36.6692
Cola micrantha	BGT5659	Cvetkovic et al. 2021	Sterculioideae	161906	90247	25606	20447	36.73551
Firmiana calcarea	NC_061661	Genbank	Sterculioideae	161263	90141	25549	20024	36.87269
Firmiana colorata	NC_054165	Genbank	Sterculioideae	162135	89573	26648	19266	37.10241
Firmiana danxiaensis	NC_057288	Genbank	Sterculioideae	161205	90114	25517	20057	36.87851
Firmiana hainanensis	NC_065869	Genbank	Sterculioideae	161031	89968	25521	20021	36.91463
Firmiana kwangsiensis	MN338197	Genbank	Sterculioideae	160836	89700	25583	19970	37.03835
Firmiana major	NC_037242	Genbank	Sterculioideae	161302	90178	25543	20038	36.86625
Firmiana pulcherrima	NC_036395	Genbank	Sterculioideae	159556	88444	25576	19960	37.12803
Firmiana simplex	NC_041438	Genbank	Sterculioideae	161268	90146	25549	20024	36.87216
Heritiera angustata	NC_037784	Genbank	Sterculioideae	168953	89054	34491	10917	36.79722
Heritiera fomes	NC_043924	Genbank	Sterculioideae	168904	88992	34496	10920	36.82506
Heritiera javanica	NC_057264	Genbank	Sterculioideae	161419	89334	26427	19231	37.01485
Heritiera littoralis	NC_043923	Genbank	Sterculioideae	168778	88926	34486	10880	36.81641
Heritiera parvifolia	NC_038057	Genbank	Sterculioideae	160193	89053	25588	19964	37.07965
Scaphium affine	GWHBHLF01000000	CGIR	Sterculioideae	161407	90087	25468	20384	36.83421
Sterculia lanceifolia	MZ901905	Genbank	Sterculioideae	160287	89355	25646	19640	36.96744
Sterculia lanceolata	NC_054170	Genbank	Sterculioideae	161118	89520	25390	20818	36.97166
Sterculia micrantha	MZ901907	Genbank	Sterculioideae	160289	89357	25646	19640	36.96698
Sterculia monosperma	NC_053571	Genbank	Sterculioideae	160178	89553	25464	19697	37.01008
Sterculia nobilis	NC_063575	Genbank	Sterculioideae	160177	89552	25464	19697	37.00906
Craigia yunnanensis	NC_045284	Genbank	Tilioideae	163166	91580	25573	20440	36.43345
Tilia americana	NC_065064	Genbank	Tilioideae	162715	91205	25571	20368	36.48281
Tilia amurensis	NC_028588	Genbank	Tilioideae	162715	91124	25597	20397	36.47666
Tilia cordata	NC_065062	Genbank	Tilioideae	162855	91164	25655	20381	36.47601
Tilia endochrysea	OK624380	Genbank	Tilioideae	162838	91264	25580	20414	36.45157
Tilia mandshurica	NC_028589	Genbank	Tilioideae	162796	91127	25649	20371	36.49168
Tilia miqueliana	NC_060401	Genbank	Tilioideae	162526	91097	25523	20383	36.52093
Tilia mongolica	NC_057237	Genbank	Tilioideae	162804	91255	25597	20355	36.45918
Tilia oliveri	NC_028590	Genbank	Tilioideae	162734	91095	25629	20381	36.49207
Tilia paucicostata	NC_028591	Genbank	Tilioideae	162653	91139	25567	20380	36.50163
Tilia taishanensis	NC_051557	Genbank	Tilioideae	162803	91114	25655	20379	36.4852
Tilia tomentosa	NC_065063	Genbank	Tilioideae	162746	91055	25655	20381	36.48876
Tilia tuan	MW800934	Genbank	Tilioideae	162318	90791	25597	20333	36.55725
Dipterocarpus turbinatus	NC_046842	Genbank		152279	83862	24101	20215	37.29996
Parashorea chinensis	NC_046579	Genbank		152002	84092	23947	20016	37.08964
Vatica odorata	NC_054172	Genbank		151553	83627	23915	20096	37.16654

Cvetkovic, T., F. Areces-Berazain, D. D. Hinsinger, D. C. Thomas, J. J. Wieringa, S. K. Ganesan, and J. S. Strijk. 2021. Phylogenomics resolves deep subfamilial relationships in Malvaceae s.l. G3: Genes, Genomes, Genetics 11.
