# Supplementary material for: The complete chloroplast genome of Diplodiscus trichospermus and phylogenetic position of Brownlowioideae within Malvaceae
Source: BMC Genomics. 2023 Sep 26;24:571. doi: 10.1186/s12864-023-09680-z (PMC10521492; doi:10.1186/s12864-023-09680-z)
Supplement: Supplementary file 3 — Additional file 3. The conserved sequences shared among subfamilies. The blue color indicated the sequences located in the IRs. [file 12864_2023_9680_MOESM3_ESM.pdf]

Additional file 3 The conserved sequences shared among subfamilies. The blue color indicated the sequences located in the IRs.

subfamily	completely conservation sequences	length (bp)	location
Bombacoideae/Malvoideae	TTATCCATATATTATCCATATA	22	ycf2-trnL
	TTAGTTCCGATCCCGATAAAGAAA	24	ycf3
	GTAGGTGTAACTGTTGAGTTCTATGGCGGCGAACT	35	psbB
	GGATTTATTCAATCACATAGTTTGGGCTCCTAGGATATGGCGCCCTTGGGGCTTTCTATTTGATTGT ATCGAAAGGCC	78	ycf2
Dombeyoideae/Tilioideae	TATTGTAAATGACTTTCT	18	ycf1
	AAAACCATTTTTTGATTCT	19	ycf1
	GGGTTTGTTCCGCCATCCTACCC	23	rpl16
	TTCTTTCTTCTTCTTACTTGTCGATGTATAACCACGTATCAT	42	psbE-petL
Byttnerioideae/Grewioideae	CATGAAATTTTATCCA	16	rpoB-trnC
	AAAGATTCCTGCGAAA	16	atpB-rbcL
	AATTTTCTATTTCGAT	16	petN-psbM
	ACTCTGGAGTTTTGTTT	17	trnT-psbD
	CCTCATTTTCCAACAAAA	18	matK
	CTTAAATTTAATCCAAGG	18	petG-trnW
	CAAATTGCCTGAGAAAAATG	20	trnT-trnL
	ATTCTTTTATTTTAATTCAA	20	ndhG-ndhI
	AACTGAAGATTAGTTTCTTA	20	trnF-ndhJ
	GAGCAATAATCAATTTTCGC	20	rps3
	ATTTTGAAATGCTTTTCTACT	21	rpoA
	ATTTAATATCCCTGATGTGTC	21	trnN-ndhF
	GACACATCAGGGATATTAAAT	21	trnN-ndhF
	TTTTGTAATGAAAGACTCAAC	21	psbK-psbI
	AATGATGAACAAATATGGATG	21	clpP-psbB
	TCTTTTTAGTTCATTGGTAAA	21	ycf1
	CAACTGCTCAATCAATTACTTC	22	petN-psbM
	TGCTGAAAACGAAAAGAAGTAC	22	ndhH-rps15
	TGTAAAAAAATCAAACAAATAGA	22	rps4-trnT
	TTCATTTTTCAAAAACATCATCAG	24	petA-psbJ
	AATTTTCCATTTATCACTCAACGA	24	rps14
	AATTAGATTGGATAGCGGGAGTGTC	25	trnC-petN
	CGAATTTTGATATAATCAACATGTTC	26	rps8-rpl14
	GGGATCCTCACGGACGGAAAAAGATT	26	ycf2
	AATCTTTTTCCGTCCGTGAGGATCCC	26	ycf2
	TTTACCTACTCAGTTTTTCTTTGAGGC	27	ndhI
	AAATCGAATAGAATATTCATTAATCAAAT	29	trnL
	ATTATATTCCATCCAGATCCCAATTCCATTCATTT	35	trnN-ndhF
	AAATGAATGGAATTGGGATCTGGATGGAATATAAT	35	trnN-ndhF
	CCAGGCCCGTCACAAGGGAAACGAAAACCAAGATTTGC	38	psaA
	GCTGTTCTTATTTTACCGGAGGGGTTTGAATTAGCCCCACC	41	petA

	GTCTTGTTCTTATTCGAAACGCCTCGTGATCTTTAACCAATT	42	ycf3
	CTGTGCCTCAACTATATCAACTGTACTTGAACTGTTAGATAATTATAGTCGA	52	ndhA
	TGGTGGATCTCTCTCTCATTTAATAAAAGTCTGAAATCTTGGGTTACTAATT	52	cemA
	GCAATGCCTATTGTGTCGGCTTGACCTTTCATAAGTGGAGACAGAATAAAGCG	53	rpoA
	ACCAATACAAGCATAGGAAATAGCATAGTATTATCCGATTCATGAGGATAAGAAAA	56	ndhF
	ATAGAAAAATATTGAAAGGGAAAAATACTTCGAAGATGAACCTGTTCCCATGCAAT	56	rpoB
	ATTTATAATTCTTCCGTTTTACTGGATGGAATTTCAATGAATTAGATTCACAAGAACCA	59	psbH
	AACCTCATTATTATAAATCAAGTTATTGAATATTTCGAACAAATCTTGAAATTCGAAACTTCCCGT<br>TATCCAATAAAAACCTA	83	ndhF
	GCATGAAAGCCCAACTACATTGCATTGCCAGAATCCATGTTGTATATTTGAAAGAGGTTGACCTC<br>CTTGCTTCTCTCATGGTACAATCCTCTTCCCGCTGAGCCCCC	107	rps12-trnV
	AGAAAGGGGGCTCAGCGGGAAGAGGATTGTACCATGAGAGAAGCAAGGAGGTCAACCTCTTTCA<br>AATATACAACATGGATTCTGGCAATGCAATGTAGTTGGGCTTTCATGC	112	rps12-trnV
	CCCTCTAATTCTTTAAGAGGTTTATCTAAAAGATTCGCGATATAACTAGGAAGACGTTTCAAATA<br>CCATACATGAGTTACTGGACATGCCAGTTTTATGTATCCCATTTGATATCTTCGTATCCG	125	rpoC1
	TTATCAAGAAGTGTATCCACGGCTTCTTGTACCAATTTTTCCTGACACATTACTAATTCCCCTGGC<br>GTAGATCTACTTGTTGTTAATAGATCGGTAAGAGTATTATTTCGATAGATAACTCTTCTATAGAGT<br>TCATTAATATCTGAACTCATTAGTTTACCCCCATCTATCTGAATGATCGG	182	rpoC1
Brownlowioideae/<br>Dombeyoideae/<br>Tilioideae	CCCAATTTATGTCCTACCATACGATCTGTTATATAAATAGGCAAATG	47	rps19
	GAAAGGTATAACTTGTACAACTCTTTCCAGTTTGCAATTCGATCCGATCCATTAGTTCGTAGAGC<br>TATTTACTCGATTGCAGACATTTCTGGAACACCTCTAAC	104	ycf2
Malvoideae/Bombacoideae/<br>Brownlowioideae/Dombeyoideae/<br>Sterculioideae/Tilioideae	CTAATCTGTAAAATAA	16	rpoC1
	AAAGCCCCCAGACGGCGTATTTTTTTGAAACGAGGCCCTCGGTAACGTGACATAAGGACTCCTTA	65	rps4
Bombacoideae/Malvoideae/<br>Brownlowioideae/<br>Dombeyoideae/Tilioideae<br>Sterculioideae/Helicteroideae/	AATACAGGATGTCCC	15	rpoC1
	AGAAGATTCGGATAAAA	17	rpoC2
	CCATCTCTTCTCGAGAGGCAA	21	ycf2
	AATCCTGCGGATACATATAATT	22	rpoB
	GCCATTTTCATTGTTACTGTTCC	23	ndhK
	GCTATAAATTCGGCAAGAATATTAGGGTG	29	rps3
	GATGGTTATATGGGTATCCAAAGTACGAACAAGATGGA	38	ycf3
	AAGGGGTATTTCCATGGGTTTGCCTTGGTATCGTGTTCATACTGTCGTATTGAATGATCCCGG	63	psbB
