# Supplementary figures and images for: The complete chloroplast genome of Diplodiscus trichospermus and phylogenetic position of Brownlowioideae within Malvaceae
Source: BMC Genomics. 2023 Sep 26;24:571. doi: 10.1186/s12864-023-09680-z (PMC10521492; doi:10.1186/s12864-023-09680-z)

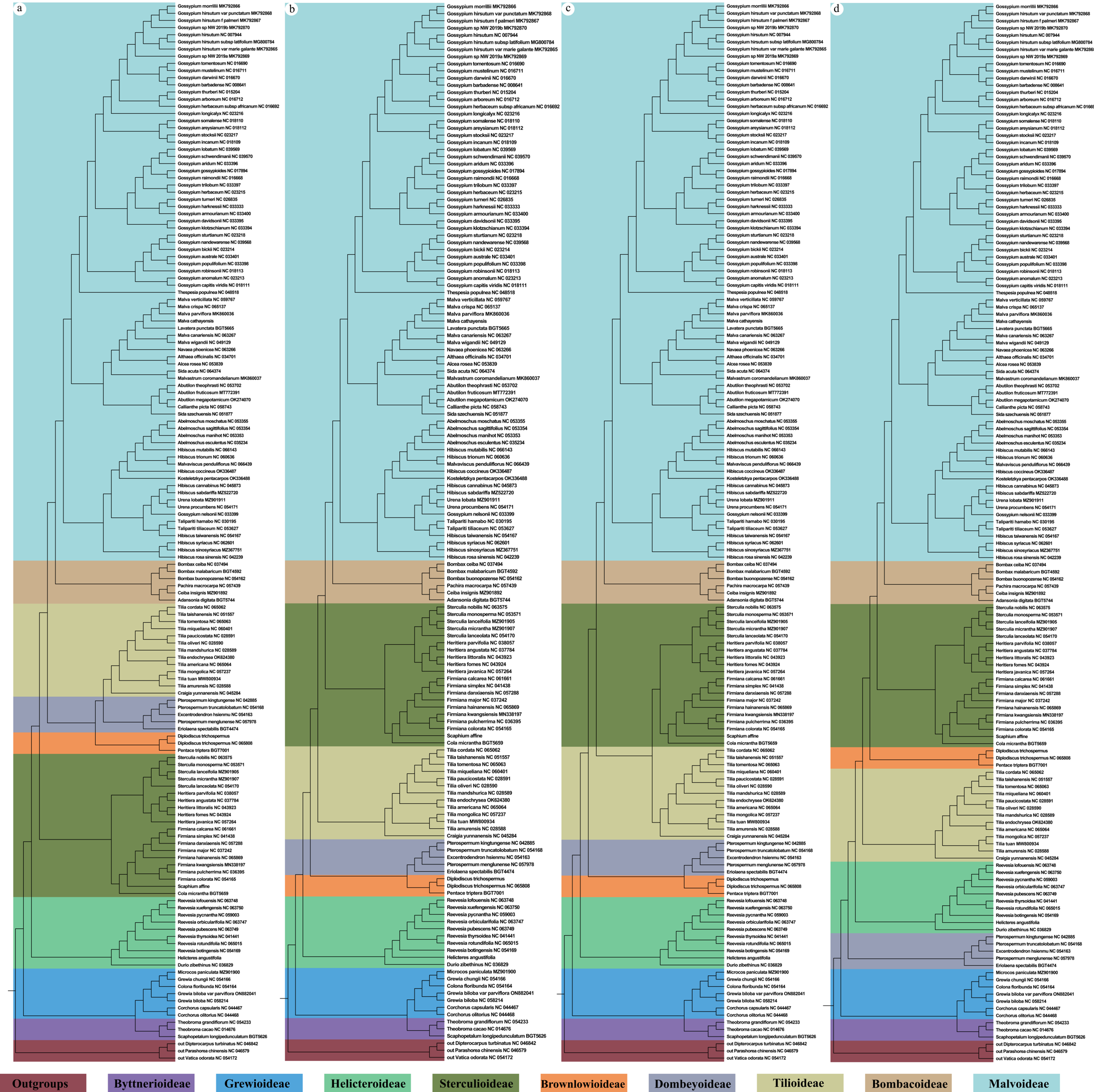

Supplement: Supplementary file 4 — Additional file 4. The hypothetical tree topologies generated by TreeGraph 2. a Helicteroideae located at the most basal position and Brownlowioideae formed a sister to the clade comprising Tilioideae and Dombeyoideae (present study); b Brownlowioideae and Dombeyoideae formed a sister group and Sterculioideae was close to Malvatheca; c Sterculioideae and Tilioideae formed a close clade which was sister to Malvatheca; d Dombeyoideae formed the earliest divergent clade. [file 12864_2023_9680_MOESM4_ESM.pdf]
